# Health-Related Quality of Life (HRQoL) of People over 65 Years of Age

**DOI:** 10.3390/ijerph19020625

**Published:** 2022-01-06

**Authors:** Marlena Krawczyk-Suszek, Andrzej Kleinrok

**Affiliations:** Department of Physiotherapy, Medical College, University of Information Technology and Management in Rzeszow, 35-225 Rzeszow, Poland; akleinrok@wsiz.edu.pl

**Keywords:** health-related quality of life, SF-36, senior, health, well–being

## Abstract

Quality of life is an important indicator of the treatment process, lifestyle, and influence of many other factors, both exogenous and endogenous, on the body. Determining the quality of life of healthy people (health-related quality of life (HRQoL), considering the influence of various factors, is important due to the possibility of making subsequent comparative analyses regarding the quality of life of people diagnosed with diseases. In addition, it allows us to identify the most crucial factors influencing the HRQoL in the process of “good aging”. The purpose of the study was to present the HRQoL level of healthy people over 65 years of age. HRQoL was measured in five-year age groups (66–70, 71–75, 76–80, >80 years), considering the analyzed factors. Finally, 1038 healthy people were included in the study. The inclusion criteria were as follows: no diagnosed chronic diseases, no permanent treatment in specialist clinics, and no constant administration of medicaments. A comparative analysis was carried out, assuming a 5% conclusion error. The SF-36 questionnaire assessing the main dimensions of the quality of life was the tool used in the study to assess the HRQoL: the physical component summary (PCS), mental component summary (MCS) and index of life quality (ILQ). The factors significantly differentiating the average level of HRQoL were as follows: gender, place of residence, education, employment status, smoking and physical activity. Relationship status (*p* > 0.05) was one of the analyzed factors that did not influence the differences in the average level of the perceived HRQoL. More than a twofold greater chance of a higher HRQoL was reported in the group of men under 75 years of age (66–70: OR = 2.01; 71–75: OR = 2.52) compared to the group of women. The same relationship was noted in the case of higher education in respondents up to the age of 80 (66–70: OR = 1.56; 71–75: OR = 2.16; 76–80: OR = 2.74). Smoking by people over 80 years of age significantly increased the chances of a higher HRQoL in each of the dimensions (PCS: OR = 4.09; MCS: OR = 12.64; ILQ: OR = 5.79). Age as a non-modifiable factor significantly differentiates the level of the HRQoL of healthy people over 65 years of age. The results of the conducted study on HRQoL can be helpful when comparing the HRQoL of healthy people with a group of people with chronic diseases.

## 1. Introduction

### 1.1. The Concept of Health in the Population of People over 65 Years of Age 

The concept of health is a subjective term, interpreted differently by each person. According to the WHO definition, “Health is a state of complete physical, mental and social well-being and not merely the absence of disease or infirmity” [1]. In the literature, one can find a number of publications analyzing the relationship between the health condition and the quality of life in groups of sick people [2,3,4,5]. However, there are no complete studies on the HRQoL of healthy people over 60 years of age. In particular, the concept of health has a different definition in the case of people over 65 years of age. This is especially due to the fact that the method of perceiving one’s own health and reacting to the factors related to it reflect the repeatedly researched concept of health-related quality of life (HRQoL) [6]. The statistical data of the world’s population shows a systematic increase in the percentage of older adults within the total number of inhabitants, which is a consequence of the extension of life expectancy, inter alia, through the progress of medicine. In Poland, there is a similarly threefold increase in the percentage of people over 60 years of age and a more than fourfold increase in people over 80 years of age, which is called the "double" aging phenomenon [7]. The sense of health in old age can be, inter alia, related to duration of life (life expectancy can be a sign of health), well-being (no pain, mental discomfort, etc.), the ability to meet social roles, i.e., "having social and economic status", the ability to meet one’s own basic needs, etc. [8]. From the perspective of society, it is important to assess the health of older adults, not only in terms of those suffering from chronic diseases and undergoing regular treatment but also to assess the quality of life of healthy people over 65 years of age. The sense of health after the age of 65 depends on the physiological process of aging and changing demographic conditions, the carried-out profession, exclusion, etc. Age, as a non-modifiable factor, increases the risk of diseases. For this reason, the percentage of healthy people decreases with increasing age, which is also associated with lowering the sense of health and quality of life. Such a dependence is confirmed by many studies, as well as data from the Central Statistical Office [9,10].

### 1.2. Health-Related Quality of Life and the Factors Determining It

In the last decade, the HRQoL and the quality of life became a crucial point of observation in medical science, analyzing epidemiological changes as well, although, for many years, biomedical results have been the main focus of the analysis, excluding QoL indicators [11]. The QoLis an important predictor of prognostic significance [12]. It is used to identify those problems most often associated with dysfunction, in either the physical or mental area. In addition, it is an important determinant of the success of the treatment process [13], while measuring HRQoL in older-adult groups can be helpful in developing and monitoring health policy [14] and political initiatives, as part of the “Age-friendly Environments in Europe” (AFEE) project [15]. The WHO gives the definition of QoL as: “An individual’s perception of their position in life, in the context of the culture in which they live and in relation to their goals, expectations, standards and concerns” [16]. However, there are many definitions that focus on different dimensions, e.g., medical, social, etc. There are also many tools for assessing the quality of life in common use, often dedicated to selected patient groups or social groups. One of them is the SF-36 questionnaire, used to assess the HRQoL of people without considering specific groups of respondents. The questionnaire contains 36 questions, and, in the summary of the analysis, it provides an overall assessment of the physical and mental dimensions of quality of life and indicates the overall index of life quality (ILQ). The assessment of individual dimensions of the quality of life is important due to the wide range of predictors influencing the quality of life as a whole [17].

The literature on the subject confirms significant correlations of socio-demographic factors, such as gender, age, place of residence, etc.—factors related to the lifestyle and the level of quality of life [18,19]. However, most studies describe analyses conducted with cohorts of sick people.

The purpose of this study was to assess the health-related quality of life (HRQoL) level of healthy people, analyze selected sociodemographic factors affecting a higher HRQoL, and give an assessment of SF-36 dimensions/spheres in the studied age groups.

## 2. Materials and Methods

### 2.1. Organization of the Study 

The study was carried out randomly on a group of healthy people in the Polish population. The study was conducted in a group of 6348 healthy people aged over 18 years old. The study was conducted, both in the form of an online survey via internet portals, as well as in the form of a direct interview with the respondent and completing a paper questionnaire, in March 2019–June 2021. The sample size, for a group over 65 years of age, that was required to obtain the power test value of 0.90 was assessed on the basis of the conducted pilot studies in a group of 220 people who are over 65 years of age. Assuming that the hypothesis Mi1 = Mi2 (ratio of the mean with regard to gender), Es = 0.59, *p* ≤ 0.05 and the power equal to 0.90, it was calculated that the number of people required in the group was 237.

**Figure 1:**
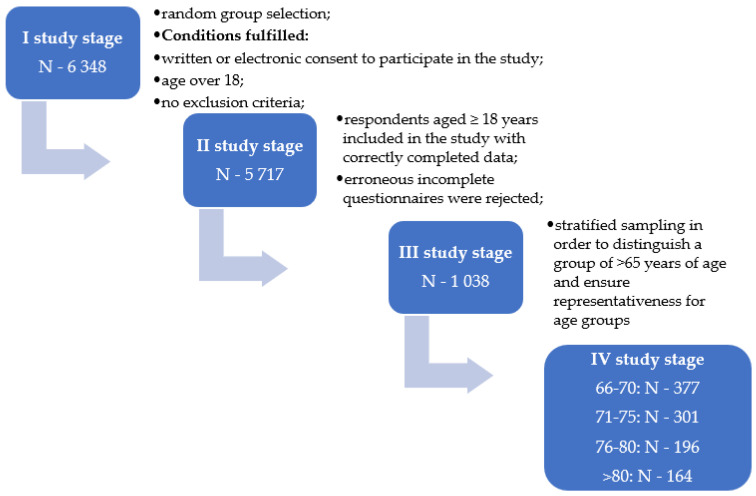
Flowchart of the participant selection process.

In order to separate a group of people who were over 65 years of age in the general database, stratified sampling was used. The criteria for inclusion in the study were as follows: age over 65 years old, no constantly administered medicaments, no diagnosed chronic diseases, and no treatments in specialist clinics. In total, 1038 healthy people were included in the study. They completed the survey and the SF-36 quality of life questionnaire. The exact flow of people is shown in the flow diagram in Figure 1. The respondents were informed about the anonymity of the conducted study, the lack of risk, and the possibility of withdrawing from participation in the study at every stage. The respondents provided their consent (in paper or electronic form) to participate in the study.

The study was performed in accordance with the Helsinki Declaration (WMA Declaration of Helsinki Ethical Principles for Medical Research Involving Human Subjects) [20].

### 2.2. Study Group

Finally, a group of 1038 people over 65 years of age was included in the study. The study respondents comprised 635 women (61.2%) and 403 men (38.8%). The average age of the studied people was 73.7 ± 5.9 years old. The number of people in each age group reflects the proportion in the Polish population of people over 65 years of age [19]. The general characteristics of the study group are presented in Table 1.

### 2.3. Questionnaire SF-36 and Sociodemographic Factors 

The study was carried out using the own-structured questionnaire, supplemented with a standardized tool to assess the quality of life, SF-36 v.2. A license to use the SF-36 questionnaire was obtained (license number: QM039882). In the analysis of data from the SF-36v.2 quality of life questionnaire, the quality of life was assessed in terms of the following dimensions: physical functioning—PF; role: physical—RP; bodily pain—BP; general health—GH; vitality—VT; social functioning—SF; role: emotional—RE; mental health—MH. Questionnaire SF-36v.2 contains one unspecified question (question 2) regarding changes in the general, subjective health of respondents during the last year (4 points) [17].

The individual parameters were combined into groups, adding four parameters concerning the assessment of the physical sphere of the quality of life and four parameters of the mental sphere. The following assignment was made: PF + RF + BT + GH + subjective sense of change in health = physical component summary (PCS); VT + SF + RE + MH = mental component summary (MCS). Both of these dimensions constitute the index of life quality (ILQ). In the Polish adaptation of the SF-36 tool, point values were used. The higher the point value in the analyzed scale, the lower the level of the perceived quality of life of the patient being studied. The maximum number of points to be obtained, according to the key proposed by Professor J. Tylka, is 103 points for PCS, 68 points for PCS, and the maximum total number of points that can be obtained, after summing up the aforementioned components, is 171 points—defined as the ILQ index [17,21].

Conducting this study, a key to analyze the SF-36 tool was used, in line with the Polish adaptation of the tool conducted by Prof. Tylka, due to the fact that the study was conducted in Poland. Adaptation of the SF-36 questionnaire to Polish conditions introduces point values to the assessment of individual spheres and dimensions analyzed in the questionnaire, while in the original version, the values obtained in the questionnaire are given in percentage units. In the analysis of the scores of the SF-36 questionnaire, according to the key created by Prof. Tylka, as previously mentioned, the fewer the negative assessments (complaints, etc.) of the examined person, the better the level of HRQoL [17,21]. On the other hand, analysis of the percentages shows a proportional relationship between the percentages and the quality of life, which means that the higher the percentage, the better the quality of life. The methods of coding and interpretation of the SF-36 questionnaire in the Polish adaptation and in the original key are presented in Appendix A.

The SF-36 tool was used to analyze the health-related quality of life (HRQoL) in a group of healthy people. The reliability of the standardized tool used to assess the analyzed feature—HRQoL—was assessed, with the applied score being in accordance with the Polish adaptation of the tool mentioned above. The mean point values and standard deviation with confidence interval were indicated, as well as the range of values and the upper and lower quartile for individual spheres and dimensions for the entire study group. The value of the Cronbach’s alpha (α) reliability coefficient, assessing the correlations of variables in their own dimensions (internal consistency), was above 0.80 for the analyzed spheres and dimensions of HRQoL. The value of the Cronbach’s α coefficient indicates a high homogeneity of the SF-36 questionnaire used for the following analysis. The Cronbach’s α coefficient is a cumulative measure, generating a value from 0 to 1 for each dimension. Values higher than 0.7 are considered acceptable [22] (Table 2). 

Quality of life and health-related quality of life (HRQoL) are very often the main goals of the study, especially in a group of people with disease entities or an HRQoL correlated with numerous factors, e.g., physical activity in a group of healthy people [23]. The literature indicates many standardized tools for assessing the overall quality of life and health-related quality of life. Short form 36 (SF-36) is one of the most frequently used tools in randomized studies and a significant health assessment [24,25,26]. The SF-36 was translated into many languages [27], or the tool was adapted to the conditions in the country where the tool is used in studies, as is the case in Poland [21]. The results of studies conducted with the use of the SF-36 tool are still quite widely published in reviewed magazines [4,28,29]. One study found that 33.3% of the 63 articles included in the analysis were using SF-36 for QoL assessment [30]. The SF-36 questionnaire was also the most widely used questionnaire in groups of older adults. The internal consistency of the tool for the group of people aged 65–104, its reliability measured by the Cronbach alpha coefficient, exceeded 0.80 in all dimensions except for social functioning (analysis of the tool being made in percentages, according to the original SF-36 coding). The assessment of the reliability of the tool, according to calculations compliant with the Polish adaptation of the tool, confirms a satisfactory level of the indicator of >0.80 in each of the measurements and spheres of the SF-36 questionnaire. The reliability and high homogeneity of SF-36 components in the group of older adults, as indicated above, allows the widespread use of the tool for study in groups of people over 65 years of age.

The following levels were determined by examining social and demographic factors. In the case of relationship analysis, the fact of being in a relationship or being single was analyzed. Being in a relationship meant having a husband/wife or a partner; being single meant not being in a relationship. The smoking habit variable was a dichotomous variable with yes-no response levels. A negative answer meant no addiction. Passive smokers were not included in the analysis. Physical activity was a dichotomous variable: yes or no. The authors defined physical activity, based on the definition of physical activity presented by the WHO. According to this definition and WHO recommendations, older adults should be physically active at least 3 times per week. Training should be aimed at complex exercises involving balance and muscle-strengthening exercises with moderate or higher intensity, to reduce the risk of future falls and increase physical performance [31]. The fulfillment of these assumptions was tantamount to giving an affirmative answer to the question regarding physical activity.

### 2.4. Statistical Analysis 

Only fully entered data were included in the analysis. A lack of data in a respondent’s answers was tantamount to the rejection of the given questionnaire.

The normality of the distribution of the measurable variable was checked using the W Shapiro–Wilk test. The non-compliance of the distribution of variables with the normal distribution confirmed the validity of the application of non-parametric analysis. A comparative analysis of the differences between the two groups was carried out using the Mann–Whitney U test, whereas comparative analysis of more than two groups was carried out using the Kruskal–Wallis test. Multiple comparisons were used to present the *p*-value for comparisons of the levels of the age groups variable in individual spheres and dimensions of SF-36. For the purposes of the analysis, the study group was divided into 4 age groups: 66–70 years, 71–75 years, 76–80 years and >80 years. When assessing the HRQoL of the older adults, the first inclusion criterion was an age above 65 years. The average values in individual dimensions and spheres were assessed, considering age groups. Replacing the points obtained in the SF-36 questionnaire with percentage values will allow for an objective assessment of the differences in HRQoL for individual age groups, and the reference of these values to the dimensions and spheres compared in the age groups. Percentage analysis was carried out in each of the spheres of the SF-36 quality of life questionnaire, considering the intergroup differences in points. The data were prepared for analysis as follows. The difference of point values for the compared groups was calculated according to the formula: $\left|\bar{x_{1}}-\bar{x_{2}}\right|$. The results indicate the absolute point value of the difference. In the case of more than two groups, the highest and the lowest point values were included in the calculation of the difference, which is marked as follows: $\left|\bar{x_{MAX}}-\bar{x_{MIN}}\right|$. The percentage of the difference from the total (%DG) was calculated according to the formula:

$$\%\mathrm{DG}=\frac{\left|\bar{x_{1}}-\bar{x_{2}}\right|}{\sum \mathrm{SF}-36\;\mathrm{in}\;\mathrm{dimensions}}*100\%$$

The absolute point value of the difference in the quality-of-life level in the compared groups was divided by the sum of points specific for the analyzed sphere of the SF-36 questionnaire (∑SF-36 in dimensions). Then, the result was multiplied by 100%, finally obtaining the percentage value of the difference between the analyzed groups in relation to the total points in the analyzed sphere of SF-36. The data are presented in Section 3.

Spearman rank r correlation coefficients were indicated to assess the correlation between the spheres and dimensions of SF-36, by age group and within age groups. Logistic regression was carried out, assessing the chances of a higher quality of life, considering the analyzed factors. The odds ratio (OR) and the 95% confidence interval (−95%CI +95%CI) were presented. For the purposes of the analysis, the following division of the quantitative variable was made: a higher level of quality of life is the percentage of points obtained in individual dimensions (PCS, MCS, ILQ) equal to 50% and lower. This meant that obtaining a lower number of points, in line with the Polish adaptation of the tool, indicated higher quality of life. The odds ratio (OR) and the 95% confidence interval (−95%CI; +95%CI) were presented. Using percentages in variable transformation (PCS, MCS and ILQ) made it possible to correctly assess the level of HRQoL analyzed in the regression analysis and simplify the variable replacement procedures.

Statistical dependences were considered significant if their level of significance was *p* < 0.05.

## 3. Results

The mean values and standard deviations were indicated, along with confidence interval values, for each of the SF-36 spheres and dimensions, considering age groups. The indicated values in Table 3 refer to the point values obtained in SF-36, in accordance with the Polish adaptation of the tool.

The mean value of points in all spheres and dimensions of SF-36 increased in subsequent age groups. The exact data are presented in Table 3.

The analysis of the influence of particular sociodemographic variables on HRQoL was carried out in the following spheres: PCS, MCS and ILQ. According to the SF-36 tool key, the higher the point value, the lower the perceived level of HRQoL. In the group of people between 66 and 70 years of age, a lower HRQoL in the physical sphere (PCS) was reported by women (*p* = 0.003), in the group of people living in rural areas (*p* = 0.011), and by people with only primary education (*p* = 0.001). Significant differences in the MCS dimension were noted at individual levels of education—higher: 21.3 ± 10.6 points, secondary: 23.0 ± 10.2 points, and elementary: 25.1 ± 9.1 points (*p* = 0.032); the higher the education level, the higher the quality of life. A higher HRQoL in MCS was also noted in the group of working people (16.6 ± 9.2 points) compared to those who were retired (24.0 ± 10.1 points), pensioners with a disability (24.2 ± 9.5 points), or non-working people (23.8 ± 6.9 points) (*p* = 0.001). The ILQ index was significantly higher in the group of women (*p* = 0.015), people living in rural areas (*p* = 0.018), people with only primary education (*p* = 0.002), and among retirees (*p* = 0.003), which confirmed a lower HRQoL level in the groups mentioned.

In the group of people aged 71 to 75, a significantly lower level of HRQoL in terms of PCS was recorded in the group of people living in rural areas (*p* = 0.001), people with only primary education (*p* < 0.001), among pensioners (*p* = 0.028), and in smokers (*p* = 0.029). In the MCS dimension, a lower HRQoL was found in the group of women (*p* < 0.001), people living in rural areas (*p* = 0.003), and people with primary education (*p* < 0.001). The overall ILQ index indicated a lower HRQoL in women (*p* = 0.010), people living in rural areas (*p* = 0.001), people with only primary education (*p* < 0.001) and in the group of people with disabilities (*p* = 0.023).

In the 76–80 age group, there were significant differences in all analyzed dimensions: PCS, MCS and ILQ were reported in the education level analysis. People with lower education (secondary: PCS: 55.2 ± 20.2 points; MCS: 25.4 ± 10.8 points; ILQ: 80.6 ± 29.0 points; elementary: PCS: 65.1 ± 18.6 points; MCS: 28.1 ± 9.6 points; ILQ: 93.3 ± 25.9 points) assessed their HRQoL significantly lower than those declaring a higher level of education (PCS: 41.4 ± 17.8 points; MCS: 19.0 ± 9.1 points; ILQ: 60.4 ± 24.7 points) (*p* < 0.001).

In the group of the oldest respondents, a significantly lower HRQoL was recorded among non-smokers. However, there are only 21 smokers in the group of smokers (12.8% of the group). The same tendency was observed in the youngest study group, but no significant differences were found (*p* > 0.05).

Lack of physical activity in all studied people in each of the analyzed age groups, and in each of the analyzed dimensions of SF-36, significantly decreased the HRQoL of the studied people (*p* < 0.05) (Table 4).

Due to the failure to meet the assumption of normal distribution, the Spearman rank correlation coefficient was determined by analyzing the correlation of SF-36 spheres and dimensions with respect to age groups (general analysis). The value of the coefficient, only in the case of the MH sphere, did not indicate a significant monotonic dependence. In the remaining analyzed levels of SF-36, significant relationships were indicated (*p* < 0.05 for SF and *p* < 0.001 for PF, RF, BP, GH, VT, RE, SCH, PCS, MCS, ILQ). Correlations at individual levels did not exceed the values of r = 0.32. The lowest values of the Spearman r coefficient and, at the same time, a significant correlation were indicated for the spheres: SF (r = 0.10), RE (r = 0.11), SCH (r = 0.15), VT (r = 0.16) versus ILQ (Table 5).

Non-parametric correlations within spheres and dimensions were conducted for the entire study group and for 4 age groups. The correlations were shown in Appendix A.

Multiple comparisons were made, indicating significant relationships between the analyzed age groups. The numerical value of the average difference between the age groups is indicated. The analysis of the relationship between 4 age groups and SF-36 was conducted using the Kruskal–Wallis test and confirmed a significant relationship between age groups and spheres: PF, RF, BP, GH, SCH, PCS, MCS and ILQ at the level of *p* < 0.001, *p* = 0.005 for the RE sphere, and no significance for the spheres: VT (*p* = 0.070), SF (*p* = 0.136) and MH (*p* = 0.346). The most significant dependencies were recorded between the age groups of 66–70 and the oldest respondents (>80 years of age). Significantly lower HRQoL in the spheres: PF, RF, BP, GH, RE, SCH, PCS, MCS, ILQ were selected by the oldest respondents participating in the study. The point differences were, respectively: −13.92, −4.84, −1.32, −1.14, −2.63, −0.31, −20.49, −5.66, −26.16 points. Between the age groups of 71–75 and 76–80, no significant relationships were found in any of the analyzed levels of SF-36. The remaining intergroup comparisons are presented in detail in Table 6.

In the group of people aged 66–70, gender, level of education and physical activity had a major influence on PCS. In the group of men, there is an almost 2.5-fold higher chance (ILQ: OR = 2.42; Cl: 1.54–3.79) of a higher HRQoL compared to the group of women. Higher education (OR = 1.64; Cl: 1.15–2.32) and physical activity (OR = 1.90; Cl: 1.19–3.04) also significantly increased the chances of a higher HRQoL in the PCS dimension. The same factors significantly increased the HRQoL in the case of the ILQ index. In the group of men, the chance of experiencing a higher HRQoL was 2-fold more frequent (ILQ: R = 2.01; Cl: 1.27–3.19). A higher level of education (OR = 1.56; Cl: 1.09–2.23) increased the chances of a higher HRQoL by almost twice. The same relationship was indicated in the case of physical activity (OR = 1.68; Cl: 1.04–2.72). In the MCS dimension of the HRQoL, men had a significantly lower chance of a higher HRQoL (OR = 0.29; Cl: 0.14–0.63).

In the group of people aged 71 to 75, higher education significantly influenced a higher HRQoL level in terms of PCS, increasing the chances of having a higher HRQoL by almost twice (OR = 1.72; Cl: 1.15–2.57), and in terms of physical activity, where the chance of occurrence was almost 3-fold higher than in the group of people not engaged in physical activity (OR = 2.73; Cl: 1.52–4.91). Men were almost 3-fold more likely to show a significantly higher HRQoL level, considering the mental sphere (OR = 2.63; Cl: 1.46–4.75). The chances of a higher value of the overall ILQ index were indicated in the case of higher education (OR = 1.82; Cl: 0.37–1.14). As far as smokers were concerned, the chance of a higher HRQoL was lower than in the group of non-smokers (OR = 0.41; Cl: 0.20–0.84). In the group of men, there was an almost 2.5-fold higher chance of a higher HRQoL compared to the group of women (OR = 2.52; Cl: 1.48–4.29). In the group of people with higher education, there was a 2-fold higher chance of a higher HRQoL (OR = 2.16; Cl: 1.41–3.31). A more than 3-fold higher chance of a higher HRQoL was recorded in the group of people practicing physical activity (OR = 3.27; Cl: 1.69–6.32).

In the group of people aged 76–80, higher education (OR = 2.07; Cl: 1.23–3.49), smoking (OR = 2.58; Cl: 1.23–3.49) and physical activity (OR = 2.93; Cl: 1.21–7.12) had a significant impact on a higher HRQoL, in terms of PCS. People with higher education had a 2-fold higher chance of a higher HRQoL in MCS (OR = 2.01; Cl: 1.04–3.88). Higher education increased the chances of a higher HRQoL almost 3-fold when assessed in the general ILQ index in the studied age group (OR = 2.74; Cl: 1.58–4.73).

In the group of people over 80 years of age, the chance of a significantly higher HRQoL in terms of PCS is lower in women compared to men (OR = 0.35; Cl: 0.12–0.99). On the other hand, smoking increased the chances of a higher HRQoL in this dimension over 4-fold (OR = 4.09; Cl: 1.27–13.19). This factor also increased the chances of a higher HRQoL in the case of MCS (OR = 12.64; Cl: 1.57–101.89) and overall ILQ (OR = 5.79; Cl: 1.89–17.70). Physical activity increased the chances of a higher HRQoL after the age of 80 almost 6-fold (OR = 5.70; Cl: 1.14–28.48) (Table 7).

## 4. Discussion

Well-being changes in every period of our lives. Needs, professional activity, the drive for success, as well as the number and scope of responsibilities, all change. On the other hand, the increase in the percentage of people over 65 years old and the popularity of the term “good aging” increase the need to study the basic sociodemographic factors influencing the health condition and, thus, the perceived HRQoL of older adults [32]. The perception of the physical and mental spheres in groups of young people differs from the perception of the same spheres in groups of people over 50 years of age. A decrease in positive assessments of the state of health in the Polish population is recorded in the statistics of the CSO from 2019. In the group of people aged 60–69, 34.9% declared that they were in good or very good health; in the group of people aged 70–79, the percentage decreased to 21.7%, while after the age of 80, the percentage was only 15.7%. However, along with the progress of civilization, there is a statistical increase in positive assessments of the quality of life in groups of people over 80 years old in 2019, compared to in 2014 (+3.5%) [9]. The author’s study shows that the HRQoL of the study group deteriorated over the years. The lowest HRQoL and, at the same time, the highest average point value was observed in the group of people over 80 years of age. The largest differences compared to the youngest group (61–65 years of age) are observed in the PCS dimension (+19.9%) (*p* < 0.001). In terms of MCS, the average result increased by 8.2%. This confirms the influence of age on the reduction of the HRQoL, despite the lack of permanent treatment due to chronic diseases. In particular, the reduction in the quality of life is noted in the physical dimension. With greater age, disability that gradually limits the human motor functions will develop over time.

In each age group, women declared a lower level of HRQoL, and between the age of 66 and 75, the differences were statistically significant (*p* < 0.05). The mean score in the PCS dimension was 51.2 ± 20.5 points in women and 45.3 ± 19.1 in men. The exception was the MCS dimension in the group of people aged 66–70, where the average difference between gender was only 0.4 points and no significant differences were found, and, in the PCS dimension, at the age of 70–75. No significant gender differences were found in the studied people over 75 years of age (*p* > 0.05). When assessing the chances of a good quality of life, such chances were much more often reported in the group of men up to 80 years of age. The data of the CSO confirm, in the subjective opinion of the respondents, worse health in the group of women compared to men [9].

The place of residence is another of the analyzed sociodemographic factors. In the authors’ study, significant differences in the compared groups occurred more often, up to the age of 75 (*p* < 0.05), and they were larger in the PCS dimension. After the age of 75, there were no significant differences in the average level of HRQoL depending on the place of residence (*p* > 0.05). The average point difference in all dimensions was lower, especially in the dimension of PCS. For this reason, it can be concluded that the average level of HRQoL in the physical dimension of people living in rural areas after 75 years of age has come close to the average level of quality of life of people living in cities. The increase in the average point value in the physical sphere after the age of 80, compared to the age group of 66–70, for people living in rural areas is 20.8 points, while for people living in cities, an increase of 24.1 points is observed, which confirms the decrease in the HRQoL over the years. When analyzing the reduced level of HRQoL in the physical dimension, the frequency of physical activity among people living in cities and rural areas should be considered. According to the data from the Central Statistical Office, 31% of the city’s inhabitants and only 18% of the rural population were physically active. Better perception of the widely understood quality of life was also indicated by other authors when studying the populations of other countries [9,32,33]. Therefore, on the basis of these data, it can be concluded that a decrease in physical activity and a deterioration in the quality of life with age are observed.

Other socio-demographic factors significantly influencing the HRQoL are education and employment. In the original studies, up to the age of 80, a higher level of education most often significantly increased the chances of a higher HRQoL, twice on average (*p* < 0.05). An average higher HRQoL was recorded in the group of people with higher education. This relationship was opposite in the group of the oldest studied people, i.e., over 80 years of age. A higher HRQoL was observed in the group of working people, while among retired people, the average point values were higher, which indicates a lower level of perceived HRQoL. The significance of the aforementioned factors in the assessment of the quality of life is also confirmed by other authors [32].

Smoking is one of the risk factors. It repeatedly contributes to disease, which is confirmed by studies among sick people exposed to this factor. In the studies, healthy smokers repeatedly indicated a higher level of HRQoL in individual age groups, in two of the analyzed dimensions (PCS, MCS) and in the ILQ index. This phenomenon can be explained by the smoker’s paradox, as described in the literature, which indicates lower mortality among smokers hospitalized due to acute coronary syndrome [34], although the results of the studies raise many doubts; the importance of the age of the studied groups was considered, as the difference between the smoking and non-smoking groups was 10 years on average. Smokers constituted a younger group, and age is a non-modifiable factor that increases the risk of diseases with increasing years of age, which leads to a reduction in the quality of life [35,36,37]. However, in the author’s studies, a significantly higher chance of a higher HRQoL in smokers was noted, especially after the age of 80. In addition, this situation can be considered in terms of the health of the individual and the lack of a major influence of smoking on physiological changes in the body. Good health at over 80 years old is also determined by the individual predispositions of the body. The psychic sphere is also a crucial factor, due to the fact that it influences the perceived level of quality of life. Owing to the fact that tobacco is an addictive substance with a proven effect on the body, it reaches the brain in 15–20 s on average. The rate of delivery to the brain is considered to be a crucial factor that magnifies the frequent use of inhaled nicotine compared to other methods of administration, and higher arterial concentrations are associated with the almost immediate psychological effects of nicotine [38]. Mood, cognitive and relaxation abilities after smoking are believed to be the result of nicotine stimulation of the presynaptic nicotinic receptors [39]. Hence, it is concluded that the feeling of a better mood as a result of smoking can contribute to an increase in the level of perceived HRQoL, especially in the group of people over 80 years old. However, the size of this group is 21 people, which constitutes 12.8% of all people aged >80 years of age.

The literature discusses the phenomenon of population aging and “frailty” as a common, complex geriatric condition characterized by disturbed physiological functions, correlated with reduced resistance to minor stressing factors [40]. Such a state of health increases the risk of mental as well as physical disability and increases the risk of falls, etc. [41,42]. It is effective and modifiable in the area of health promotion activities in the group of older adults [31]. As emphasized by the WHO, “Physical activity is important across all ages, and should be integrated into multiple settings” [43]. In each of the compared age groups, physical activity significantly increased the level of perceived HRQoL (*p* < 0.05). The chance for a better HRQoL related to physical activity improved with age in people over 80 years of age, increasing the chances of a higher HRQoL in terms of MCS even 6-fold (OR = 5.70). The influence of physical activity on the quality of life was confirmed by many studies [44]. The chances of indicating a high level of quality of life by people with low physical activity of productive age (55–64 years) were seven times lower than for people who declared physical activity (OR = 0.14). It was shown that the quality of life significantly decreases with age [45]. Other authors confirmed decreased physical activity in people over 50 years of age, which can be a risk factor significantly influencing health and the perceived level of quality of life at a later age [46,47]. It was emphasized many times that decreased physical activity with age is inevitable. However, many scientific reports confirm that seniors have the potential to maintain physical activity, especially when adjusted to individual abilities, and should avoid inactivity [48,49].

Marital status was the only factor in the author’s studies that did not significantly differentiate the analyzed groups (*p* > 0.05).

### Limitation

The presented study results should be viewed in terms of certain limitations. The relationship variable includes those people who are currently married/in a partnership or people who are not in a partner relationship. The variable should be expanded to include widowed people, and the correlation of this variable with the period of time in which they remain single should be assessed. In addition, it is worth assessing the HRQoL, correlated with passive smoking, the fact of quitting smoking, and the number of years during which a person did not smoke. In addition, the assessment of the impact of physical activity on quality of life should be expanded, especially the correlation with the dimensions of PCS and MCS, considering the amount and duration of physical activity per week.

## 5. Conclusions

Age, as an unmodified factor in healthy people over 65 years old, reduces the quality of life. A particularly low level of HRQoL in the studied group is observed among women. At the same time, the low correlation coefficient indicates the need to look for other predictors influencing HRQoL. A strong correlation between RF and BP and ILQ was confirmed in people over 65 years of age. For people over 80 years of age, mental health was an important factor influencing the HRQoL. The most visible changes in HRQoL in almost all dimensions of SF36 are visible during the transition from the seventh to the eighth life decade and are further visible over 80 years of age.

The impact of smoking on the quality of life, including smokers, currently non-smokers and passive smokers, requires an extension of the study.

Data on the quality-of-life level among healthy people can be a reference point for comparisons and discussions with the results of studies carried out in groups of people at risk of disease.

## Figures and Tables

**Table 1:** General characteristics of the study group of the respondents.

Characteristics	Age Group (*n*; %)								Total (*n* = 1038)	
	66–70 (377; 36.3)		71–75 (301; 29.0)		76–80 (196; 18.9)		>80 (164; 15.8)			
	*n*	%	*n*	%	*n*	%	*n*	%	*n*	%
Gender: female/male	206/171	54.6/45.4	172/129	57.1/42.9	148/48	75.5/24.5	109/55	66.5/33.5	635/403	61.2/38.8
Place of residence: city/village	211/166	56.0/44.0	174/127	57.8/42.2	89/107	45.4/54.6	93/71	56.7/43.3	567/471	54.6/45.4
Education: higher/secondary/elementary	78/192/ 107	20.7/50.9/28.4	44/144/113	14.6/47.8/37.5	25/83/ 88	12.8/42.3/44.9	13/61/ 90	7.9/37.2/ 54.9	160/480/398	15.4/46.2/38.3
Employment status: working/disability pension/retirement/does not work	37/42/ 280/18	9.8/11.1/74/34.8	3/15/ 277/6	1.0/5.0/ 92.0/2.0	0/12 / 177/7	0.0/6.1/ 90.3/3.6	0/7/ 151/6	0.0/4.3/ 92.1/3.7	40/76/885/37	3.8/7.3/85.3/3.6
Relationship: in relationship/single	291/86	77.2/22.8	208/93	69.1/30.9	135/61	68.9/31.1	74/90	45.1/54.9	708/330	68.2/31.8
Smoking: yes/no	64/313	17.0/83.0	47/254	15.6/84.4	22/174	11.2/88.8	21/143	12.8/87.2	154/884	14.8/85.2
Physical Activity: yes/no	139/238	36.9/63.1	72/229	23.9/76.1	32/164	16.3/83.7	17/47	10.4/89.6	260/778	25.0/75.0

**Table 2:** The reliability of the SF-36 questionnaire in the examined Polish group of healthy people and the distribution of the values of variables for the total number of respondents. Data coded in accordance with the Polish adaptation of the SF-36 tool (point values).

Spheres	∑	X (−95Cl–+95Cl)	SD (−95Cl–+95Cl)	Reference (Min–Max)	Q1	Q3	Cronbach’s Alpha (α)
PF	50	20.9 (20.1–21.7)	13.3 (12.8–13.9)	0.0–50.0	9.0	13.3	0.83
RF	20	13.4 (12.9–13.9)	8.1 (7.7–8.4)	0.0–20.0	5.0	8.1	0.84
BP	9	4.0 (3.9–4.1)	2.0 (1.9–2.1)	0.0–9.0	3.0	2.0	0.86
GH	20	10.9 (10.8–11.1)	2.3 (2.2–2.4)	3.0–18.0	10.0	2.3	0.86
VT	20	9.7 (9.5–9.8)	2.6 (2.5–2.7)	2.0–17.0	8.0	2.6	0.86
SF	8	3.2 (3.1–3.3)	1.8 (1.7–1.8)	0.0–8.0	2.0	1.8	0.86
RE	15	8.4 (8.0–8.8)	6.6 (6.3–6.9)	0.0–15.0	0.0	6.6	0.85
MH	25	10.8 (10.6–11.0)	3.2 (3.0–3.3)	3.0–20.0	9.0	3.2	0.86
SCH	4	2.2 (2.2–2.3)	0.9 (0.9–1.0)	0.0–4.0	2.0	0.9	0.86
PCS	103	56.1 (54.8–57.4)	21.4 (20.5–22.3)	7.0–101.0	41.0	21.4	0.81
MCS	68	25.3 (24.7–26.0)	10.5 (10.0–11.0)	2.0–46.0	16.0	10.5	0.83
ILQ	171	81.4 (79.6–83.2)	30.2 (29.0–31.6)	9.0–147.0	58.0	30.2	0.84

X—average; −95Cl/+95Cl—95% confidence interval of average or standard deviation; SD—standard deviation; Reference (Min–Max)—value between minimum and maximum; Q1—lower quartile; Q3—upper quartile; PF—physical functioning; RF—role: physical; BP—bodily pain; GH—general health; VT—vitality; SF—social functioning; RE—role: emotional; MH—mental health; SCH—subjective sense of change in health; PCS—physical component summary; MCS—mental component summary; ILQ SF-36—index of life quality; ∑—sum of points in individual spheres and dimensions (point value)—in accordance with the Polish adaptation of the SF-36 [17].

**Table 3:** The average level of the HRQoL in individual spheres, considering the intergroup averages (point values).

Variable	Spheres											
	PF	RF	BP	GH	VT	SF	RE	MH	SCH	PCS	MCS	ILQ
∑	50	20	9	20	20	8	15	25	4	103	68	171
66–70 (*n* = 377)												
X	15.9	11.7	3.5	10.4	9.2	3.0	7.6	10.6	2.0	48.5	23.3	71.8
−95Cl	14.7	10.8	3.3	10.1	8.9	2.8	6.9	10.3	1.9	46.5	22.2	68.9
+95Cl	17.1	12.5	3.7	10.6	9.4	3.1	8.3	10.9	2.1	50.5	24.3	74.6
SD	11.8	8.5	1.8	2.3	2.4	1.7	6.7	3.2	0.9	20.1	10.1	28.6
−95Cl	11.0	8.0	1.7	2.2	2.3	1.6	6.2	3.0	0.8	18.8	9.4	26.7
+95Cl	12.7	9.2	1.9	2.5	2.6	1.8	7.2	3.4	0.9	21.6	10.8	30.8
Me	15.0	15.0	3.0	10.0	9.0	3.0	10.0	11.0	2.0	48.0	25.0	75.0
71–75 (*n* = 301)												
X	21.9	13.2	4.1	11.3	9.7	3.3	8.5	10.9	2.4	57.3	25.6	83.0
−95Cl	20.5	12.3	3.8	11.1	9.4	3.1	7.7	10.5	2.3	55.0	24.3	79.6
+95Cl	23.3	14.2	4.3	11.5	10.1	3.5	9.2	11.2	2.5	59.6	26.9	86.4
SD	12.5	8.1	2.1	2.0	2.7	1.7	6.7	2.9	0.9	20.3	11.2	30.0
−95Cl	11.5	7.5	2.0	1.8	2.5	1.6	6.2	2.7	0.8	18.8	10.4	27.8
+95Cl	13.6	8.8	2.3	2.1	3.0	1.9	7.3	3.1	1.0	22.1	12.2	32.6
Me	23.0	20.0	4.0	11.0	10.0	3.0	10.0	11.0	2.0	57.0	28.0	83.0
76–80 (*n* = 196)												
X	21.7	14.2	4.2	11.0	9.9	3.4	8.4	11.0	2.3	57.9	25.9	83.7
−95Cl	19.9	13.1	3.9	10.6	9.5	3.1	7.5	10.5	2.1	55.0	24.4	79.6
+95Cl	23.5	15.3	4.5	11.3	10.3	3.7	9.3	11.5	2.4	60.8	27.3	87.8
SD	12.9	7.7	1.9	2.6	2.9	1.9	6.5	3.8	1.1	20.6	10.4	29.1
−95Cl	11.8	7.0	1.8	2.3	2.7	1.7	5.9	3.5	1.0	18.8	9.5	26.4
+95Cl	14.4	8.5	2.1	2.9	3.2	2.1	7.2	4.2	1.2	22.9	11.6	32.3
Me	22.0	20.0	4.0	11.0	10.0	4.0	10.0	11.0	2.0	60.5	26.5	84.0
>80 (*n* = 164)												
X	29.8	16.5	4.8	11.5	10.4	3.5	10.2	11.2	2.3	69.0	28.9	97.9
−95Cl	27.7	15.6	4.5	11.2	10.1	3.2	9.3	10.7	2.2	65.9	27.5	93.7
+95Cl	31.9	17.5	5.1	11.8	10.7	3.7	11.2	11.6	2.5	72.1	30.3	102.1
SD	13.7	6.0	2.1	1.8	2.1	1.8	6.0	2.9	0.8	19.8	9.0	27.4
−95Cl	12.3	5.4	1.9	1.6	1.9	1.6	5.4	2.6	0.7	17.9	8.1	24.7
+95Cl	15.3	6.8	2.3	2.0	2.4	2.0	6.8	3.2	0.9	22.2	10.1	30.7
Me	34.0	20.0	5.0	12.0	10.0	4.0	15.0	11.0	2.0	75.0	31.0	106.5

X—average; −95Cl/+95Cl—95% confidence interval of average or standard deviation; SD—standard deviation; Me—median; PF—physical functioning; RF—role: physical; BP—bodily pain; GH—general health; VT—vitality; SF—social functioning; RE—role: emotional; MH—mental health; SCH—subjective sense of change in health; PCS—physical component summary; MCS—mental component summary; ILQ SF-36—index of life quality; ∑—sum of points in individual spheres and dimensions (point value).

**Table 4:** Average level of the HRQoL in individual spheres, considering the intergroup averages (point values) and the differences between them.

Variable	Age Group																							
	66–70						71–75						76–80						>80					
	PCS		MCS		ILQ		PCS		MCS		ILQ		PCS		MCS		ILQ		PCS		MCS		ILQ	
	X	SD	X	SD	X	SD	X	SD	X	SD	X	SD	X	SD	X	SD	X	SD	X	SD	X	SD	X	SD
**Gender**																								
Female	51.2	20.5	23.4	9.9	74.6	28.8	59.1	18.5	27.7	10.5	86.8	27.4	58.0	21.0	26.0	10.7	84.0	29.7	67.0	20.2	28.5	9.1	95.5	27.7
Male	45.3	19.1	23.0	10.2	68.3	28.0	55.0	22.3	22.8	11.6	77.8	32.6	57.5	19.7	25.4	9.6	82.9	27.3	72.9	18.6	29.7	8.7	102.7	26.3
$\left|\bar{x_{1}}-\bar{x_{2}}\right|$	5.9	1.4	0.4	−0.3	6.3	0.8	4.1	3.8	4.9	1.1	9	5.2	0.5	1.3	0.6	1.1	1.1	2.4	5.9	1.6	1.2	0.4	7.2	1.4
%DG	5.7	-	0.6	-	3.7	-	4.0	-	7.2	-	5.3	-	0.5	-	0.9	-	0.6	-	5.7	-	1.8	-	4.2	-
*p*	0.003		0.574		0.015		0.078		<0.001		0.010		0.754		0.427		0.682		0.074		0.412		0.100	
**Place of residence**																								
City	46.2	20.4	22.4	10.5	68.6	29.6	54.2	19.9	23.9	11.4	78.1	29.7	54.8	21.6	25.2	10.9	80.1	30.5	71.0	20.5	29.6	9.0	100.5	28.3
Village	51.4	19.4	24.3	9.3	75.7	26.8	61.7	20.2	27.9	10.6	89.6	29.3	60.4	19.6	26.4	10.0	86.8	27.6	72.9	18.6	29.7	8.7	102.7	26.3
$\left|\bar{x_{1}}-\bar{x_{2}}\right|$	5.2	1	1.9	1.2	7.1	2.8	7.5	0.3	4	0.8	11.5	0.4	5.6	2	1.2	0.9	6.7	2.9	1.9	1.9	0.1	0.3	2.2	2
%DG	5.0	-	2.8	-	4.2	-	7.3	-	5.9	-	6.7	-	5.4	-	1.8	-	3.9	-	1.8	-	0.1	-	1.3	-
*p*	0.011		0.075		0.018		0.001		0.003		0.001		0.085		0.530		0.201		0.149		0.243		0.144	
**Education**																								
Higher	43.1	19.7	21.3	10.6	64.4	29.2	48.8	20.3	19.8	11.6	68.6	30.5	41.4	17.8	19.0	9.1	60.4	24.7	73.4	20.5	30.2	9.8	103.6	29.9
Secondary	47.6	20.1	23.0	10.2	70.6	28.6	54.5	18.5	25.0	10.5	79.5	27.2	55.2	20.2	25.4	10.8	80.6	29.0	66.0	21.0	28.1	9.8	94.1	29.8
Elementary	54.1	19.2	25.1	9.1	79.2	26.6	64.3	20.5	28.6	11.0	93.0	30.2	65.1	18.6	28.2	9.6	93.3	25.9	70.4	18.9	29.3	8.3	99.7	25.3
$\left|\bar{x_{MAX}}-\bar{x_{MIN}}\right|$	11	0.5	3.8	1.5	14.8	2.6	15.5	0.2	8.8	0.6	24.4	−0.3	23.7	0.8	9.2	0.5	32.9	1.2	3.0	1.6	0.9	1.5	3.9	4.6
%DG	10.7	-	5.6	-	8.7	-	15.0	-	12.9	-	14.3	-	23.0	-	13.5	-	19.2	-	2.9	-	1.3	-	2.3	-
*p*	0.001		0.032		0.002		<0.001		<0.001		<0.001		<0.001		<0.001		<0.001		0.360		0.813		0.542	
**Employment status**																								
Working	37.6	18.6	16.6	9.2	54.2	26.8	52.0	36.9	22.0	18.2	74.0	55.1	-	-	-	-	-	-	-	-	-	-	-	-
Disability pension	48.8	18.2	24.2	9.5	73.0	25.6	70.6	19.4	32.3	4.6	102.9	21.7	54.1	15.5	25.7	8.7	79.8	21.7	85.4	6.1	29.6	6.0	115.0	5.2
Retirement	49.8	20.5	24.0	10.1	73.8	29.0	56.4	20.0	25.2	11.3	81.6	29.9	57.7	20.8	25.8	10.5	83.5	29.3	68.2	19.9	28.9	9.2	97.1	27.8
Does not work	49.7	15.2	23.8	6.9	73.5	19.4	70.0	16.2	30.2	9.5	100.2	24.4	68.0	23.6	27.7	13.3	95.7	35.8	69.2	22.3	29.3	8.1	98.5	29.5
$\left|\bar{x_{MAX}}-\bar{x_{MIN}}\right|$	12.2	5.3	7.6	3.2	19.6	9.6	18.6	20.7	10.3	13.6	28.9	33.4	13.9	8.1	2	4.6	15.9	14.1	16.2	16.2	0.7	3.2	17.9	24.3
%DG	11.8	-	11.2	-	11.5	-	18.1	-	15.1	-	16.9	-	13.5	-	2.9	-	9.3	-	15.7	-	1.0	-	10.5	-
*p*	0.014		0.001		0.003		0.028		0.170		0.023		0.336		0.857		0.508		0.077		0.995		0.414	
**Relationship**																								
In a relationship	48.6	20.4	22.9	10.3	71.5	29.1	57.3	19.2	26.2	10.9	83.5	28.5	58.8	21.1	25.9	10.4	84.7	29.7	68.2	21.0	29.0	9.7	97.3	29.3
Single	48.2	19.3	24.5	9.1	72.8	26.7	57.4	22.7	24.4	11.8	81.8	33.3	55.8	19.6	25.8	10.5	81.6	27.8	69.6	18.9	28.8	8.4	98.4	25.9
$\left|\bar{x_{1}}-\bar{x_{2}}\right|$	0.4	1.1	1.6	1.2	1.3	2.4	0.1	3.5	1.8	0.9	1.7	4.8	3	1.5	0.1	0.1	3.1	1.9	1.4	2.1	0.2	1.3	1.1	3.4
%DG	0.4	-	2.4	-	0.8	-	0.1	-	2.6	-	1.0	-	2.9	-	0.1	-	1.8	-	1.4	-	0.3	-	0.6	-
*p*	0.865		0.240		0.770		0.961		0.187		0.681		0.209		0.877		0.331		0.807		0.572		0.816	
**Smoking**																								
Yes	47.1	20.4	22.5	9.9	69.6	29.1	63.7	21.7	27.9	11.8	91.6	32.2	52.1	14.5	25.4	9.4	77.5	21.0	58.8	20.3	24.1	7.4	82.9	26.9
No	48.8	20.1	23.4	10.1	72.2	28.5	56.1	19.8	25.2	11.1	81.2	29.4	58.6	21.2	25.9	10.6	84.5	29.9	70.5	19.4	29.6	9.0	100.1	26.9
$\left|\bar{x_{1}}-\bar{x_{2}}\right|$	1.7	0.3	0.9	0.2	2.6	0.6	7.6	1.9	2.7	0.7	10.4	2.8	6.5	6.7	0.5	1.2	7	8.9	11.7	0.9	5.5	1.6	17.2	0
%DG	1.7	-	1.3	-	1.5	-	7.4	-	4.0	-	6.1	-	6.3	-	0.7	-	4.1	-	11.4	-	8.1	-	10.1	-
*p*	0.458		0.466		0.466		0.029		0.087		0.054		0.052		0.544		0.098		0.038		0.033		0.033	
**Physical Activity**																								
Yes	43.5	20.8	21.8	10.3	65.3	29.7	45.9	18.6	20.3	11.3	66.2	28.6	42.5	21.5	19.2	10.8	61.7	29.7	56.6	21.2	22.9	9.3	79.5	29.5
No	51.4	19.1	24.1	9.8	75.6	27.3	60.9	19.5	27.3	10.7	88.2	28.5	60.9	19.1	27.2	9.9	88.0	27.0	70.4	19.2	29.6	8.7	100.0	26.5
$\left|\bar{x_{1}}-\bar{x_{2}}\right|$	7.9	1.7	2.3	0.5	10.3	2.4	15	0.9	7.0	0.6	22	0.1	18.4	2.4	8.0	0.9	26.3	2.7	13.8	2	6.7	0.6	20.5	3.0
%DG	7.7	-	3.4	-	6.0	-	14.6	-	10.3	-	12.9	-	17.9	-	11.8	-	15.4	-	13.4	-	9.9	-	12.0	-
*p*	<0.001		0.036		0.001		<0.001		<0.001		<0.001		<0.001		<0.001		<0.001		0.010		0.006		0.008	

X—average; SD—standard deviation; PCS—physical component summary. ∑ = 103; MCS—mental component summary. ∑ = 68; ILQ SF-36–index of life quality. ∑ = 171 points; *p*—level of statistical significance; the Mann–Whitney U test was applied if the comparison is of the two groups, and an ANOVA Kruskal–Wallis test if the comparison is of more than two groups. Intergroup analysis: $\left|\bar{x_{1}}-\bar{x_{2}}\right|$—point difference between groups for the two analyzed groups (value in points); $\left|\bar{x_{MAX}}-\bar{x_{MIN}}\right|$—point difference between the highest and the lowest value (value in points); %DG—the percentage value of the intergroup difference, in relation to the total points in the analyzed SF-36 sphere (percentage value).

**Table 5:** Spearman rank-order correlation in SF-36 by age group.

Spheres	r Spearmana	T(N-2)	*p*
PF	0.32	10.740	<0.001
RF	0.19	6.178	<0.001
BP	0.21	6.826	<0.001
GH	0.18	5.784	<0.001
VT	0.16	5.292	<0.001
SF	0.10	3.094	0.002
RE	0.11	3.600	<0.001
MH	0.05	1.528	0.127
SCH	0.15	4.989	<0.001
PCS	0.30	10.266	<0.001
MCS	0.18	5.880	<0.001
ILQ	0.27	9.200	<0.001

PF—physical functioning; RF—role: physical; BP—bodily pain; GH—general health; VT—vitality; SF—social functioning; RE—role: emotional; MH—mental health; SCH—subjective sense of change in health; PCS—physical component summary; MCS—mental component summary; ILQ SF-36—index of life quality.

**Table 6:** Intergroup comparisons and the mean value of the difference between the compared groups (SF-36 points).

Spheres	∑	Age Groups (Mean Point Difference between Age Groups)						^a^ *p*
		1 vs. 2	1 vs. 3	1 vs. 4	2 vs. 3	2 vs. 4	3 vs. 4	
PF	50	−6.06 ***	−5.85 ***	−13.92 ***	0,20 ns	−7.86***	−8.06 ***	<0.001
RF	20	−1.55 ns	−2.50 ns	−4.84 ***	−0.94 ns	−3.29 ***	−2.34 ns	<0.001
BP	9	−0.61 **	−0.71 **	−1.32 ***	−0.10 ns	−0.71 ***	−0.61 **	<0.001
GH	20	−0.93 ***	−0.60 ns	−1.14 **	0.33 ns	−0.21 ns	−0.54 ns	<0.001
VT	20	−0.56 ns	−0.71 ns	−1.22 ns	−0.15 ns	−0.67 ns	−0.51 ns	0.070
SF	8	−0.35 ns	−0.43 ns	−0.49 ns	−0.07 ns	−0.13 ns	−0.06 ns	0.136
RE	15	−0.84 ns	−0.75 ns	−2.63 **	0.09 ns	−1.79 **	−1.88 ns	0.005
MH	25	−0.30 ns	−0.43 ns	−0.60 ns	−0.13 ns	−0.30 ns	−0.17 ns	0.346
SCH	4	−0.33 **	−0.23 ns	−0.31 **	0.10 ns	0.02 ns	−0.08ns	<0.001
PCS	103	−8.84 ***	−9.38 **	−20.49 ***	−0.54 ns	−11.65 ***	−11.11 ***	<0.001
MCS	68	−2.37 ns	−2.61 ns	−5.66 ***	−0.24 ns	−3.30 ns	−3.06 ns	<0.001
ILQ	171	−11.21 **	−11.99 **	−26.16 ***	−0.78 ns	−14.95 ***	−14.17 ***	<0.001

PF—physical functioning; RF—role: physical; BP—bodily pain; GH—general health; VT—vitality; SF—social functioning; RE—role: emotional; MH—mental health; SCH—subjective sense of change in health; PCS—physical component summary; MCS—mental component summary; ILQ SF-36—index of life quality; age groups: 1—66–70 years; 2—71–75 years; 3—76–80 years; 4—>80 years; **—*p* < 0.05; ***—*p* < 0.001 *p*-value for multiple comparisons; ns—no statistical correlation for multiple comparisons; ^a^*p*—*p*-level of statistical significance—ANOVA Kruskal–Wallis, analysis between age groups.

**Table 7:** The impact of the analyzed factors on the HRQoL (PCS, MCS, ILQ) in individual age groups and the chance of a higher HRQoL among the studied people.

Age Group vs.	Factors						
	Gender	Place of Residence	Education	Employment Status	Marital Status	Smoking	Practicing Physical Activity
66–70							
PCS							
OR	2.42	1.06	1.64	0.80	0.99	1.02	1.90
−95Cl	1.54	0.66	1.15	0.57	0.57	0.57	1.19
+95Cl	3.79	1.70	2.32	1.11	1.75	1.81	3.04
*p*	< 0.001	0.816	0.006	0.176	0.985	0.956	0.007
MCS							
OR	0.29	0.66	1.06	0.73	1.27	1.90	1.58
−95Cl	0.14	0.30	0.61	0.43	0.54	0.68	0.69
+95Cl	0.63	1.42	1.87	1.23	2.98	5.34	3.62
*p*	0.002	0.284	0.830	0.230	0.585	0.219	0.278
ILQ							
OR	2.01	0.98	1.56	0.74	0.83	1.03	1.68
−95Cl	1.27	0.60	1.09	0.52	0.49	0.57	1.04
+95Cl	3.19	1.58	2.23	1.06	1.38	1.86	2.72
*p*	0.003	0.918	0.015	0.103	0.463	0.928	0.034
71–75							
PCS							
OR	1.49	0.68	1.72	0.96	1.10	0.52	2.73
−95Cl	0.89	0.40	1.15	0.43	0.63	0.25	1.52
+95Cl	2.49	1.17	2.57	2.15	1.90	1.08	4.91
*p*	0.132	0.159	0.008	0.918	0.738	0.079	0.001
MCS							
OR	2.63	0.65	1.82	1.50	1.12	0.41	1.59
−95Cl	1.46	0.37	1.15	0.68	0.62	0.20	0.78
+95Cl	4.75	1.14	2.88	3.30	2.03	0.84	3.22
*p*	0.001	0.131	0.010	0.317	0.697	0.015	0.201
ILQ							
OR	2.52	0.73	2.16	1.24	1.43	0.60	3.27
−95Cl	1.48	0.43	1.41	0.53	0.81	0.29	1.69
+95Cl	4.29	1.26	3.31	2.89	2.50	1.23	6.32
*p*	0.001	0.254	< 0.001	0.611	0.213	0.160	0.001
76–80							
PCS							
OR	1.16	1.21	2.07	1.29	1.24	2.58	2.93
−95Cl	0.55	0.61	1.23	0.46	0.64	1.01	1.21
+95Cl	2.44	2.40	3.49	3.61	2.43	6.60	7.12
*p*	0.692	0.586	0.006	0.631	0.519	0.046	0.017
MCS							
OR	2.69	1.04	2.01	0.57	0.78	2.16	2.47
−95Cl	1.00	0.47	1.04	0.17	0.36	0.58	0.66
+95Cl	7.28	2.30	3.88	1.90	1.69	8.05	9.30
*p*	0.049	0.927	0.036	0.360	0.533	0.247	0.178
ILQ							
OR	1.45	1.44	2.74	0.99	1.62	2.25	2.48
−95Cl	0.70	0.72	1.58	0.37	0.83	0.85	0.96
+95Cl	3.02	2.88	4.73	2.65	3.15	5.95	6.40
*p*	0.320	0.296	0.000	0.987	0.153	0.102	0.060
>80							
PCS							
OR	0.35	0.88	1.19	1.67	0.92	4.09	1.54
−95Cl	0.12	0.40	0.64	0.42	0.42	1.27	0.48
+95Cl	0.99	1.98	2.23	6.72	2.02	13.19	4.89
*p*	0.045	0.763	0.580	0.463	0.832	0.018	0.461
MCS							
OR	0.91	1.42	0.92	0.62	2.10	12.64	5.70
−95Cl	0.40	0.66	0.52	0.16	1.00	1.57	1.14
+95Cl	2.09	3.06	1.63	2.33	4.42	101.89	28.48
*p*	0.829	0.363	0.773	0.473	0.049	0.016	0.032
ILQ							
OR	0.43	1.54	1.14	2.13	0.65	5.79	2.65
−95Cl	0.17	0.74	0.64	0.56	0.31	1.89	0.87
+95Cl	1.07	3.22	2.05	8.13	1.33	17.70	8.08
*p*	0.067	0.243	0.654	0.264	0.234	0.002	0.084

PCS—physical component summary; MCS—mental component summary; ILQ SF-36—index of life quality; OR—odds ratio; −95Cl/+95Cl—95% confidence interval; *p*—level of statistical significance with the Wald chi-square test and logistic regression.

## Data Availability

Participants data, the full dataset and statistical code are available from the corresponding author (m.krawczyk.umlub@gmail.com). Participant consent for data sharing was not obtained but the presented data are anonymised and risk of identification is extremely low.

## Appendix A

### Appendix A.1. Materials and Methods

In the Polish adaptation, according to the key proposed by Prof. Tylka: Question 1, the following points are assigned: Excellent—0; Very good—1; Good—2; Fair—3; Poor—4 points. Question 2. Answer: Much better now than one year ago = 0 points, next 1,2,3,4,5 points. Question 3. From “a” to “j”—Answer: Yes, limited a lot = 5, Yes, limited a little = 3; No, not limited at all = 0 points. Question 4. From “a” to “d”—Answer: Yes = 5; No = 0 points. Question 5. from „a” to „c”—Answer: Yes = 5; No = 0 points. Question 6. Answer: Not at all = 0 points, next 1,2,3,4 points. Question 7. Answer: None = 0 points, next 1,2,3,4,5 points. Question 8. Answer: Not at all = 0 points, next 1,2,3,4 points. Question 9. (a,d,e,h) Answer: All the time = 0, next 1,2,3,4,5 points. Question 9. (b,c,f,g,i) Answer: All the time = 5, next 4,3,2,1,0 points. Question 10. Answer: All of the time = 4, next 3,2,1,0 points. Question 11. (a,b,d) Answer: Definitely true: 0 points, next 1,2,3,4 points. Question 11. (c) Answer: Definitely true: 4 points, next 3,2,1,0 points [17].

In the original version, Question 1 is assigned the following percentages: 1—Excellent—100%; 2—Very good—75%; 3—Good—50%; 4—Fair—25%; 5—Poor—0%. Response category: 1 = 100%, 2 = 75%, 3 = 50%, 4 = 25%, 5 = 0% relates to the questions: 1,2,20,22,34,36. Response category:1 = 0%, 2 = 50%, 3 = 100% relates to the questions: 3,4,5,6,7,8,9,10,11,12. Response category: 1 = 0%, 2 = 100% relates to the questions: 13,14,15,16,17,18,19. Response category: 1 = 100%, 2 = 80%, 3 = 60%, 4 = 40%, 5 = 20%, 6 = 0% relates to the questions: 21,23,26,27,30. Response category: 1 = 0%, 2 = 20%, 3 = 40%, 4 = 60%, 5 = 80%, 6 =100% relates to the questions: 24,25,28,29,31. Response category: 1 = 0%, 2 = 25%, 3 = 50%, 4 = 75%, 5 = 100% relates to the questions: 32,33,35 [50].

### Appendix A.2. Results

The tables below show the non-parametric correlations between the individual items of the variable and the value of Spearman’s rank correlation coefficient. Spearman r values that are not statistically significant are not shown in Table A1, Table A2, Table A3, Table A4 and Table A5. Both in the general analysis and in individual age groups, the strongest correlations with ILQ are found in the dimensions of PCS and MCS. For the whole, Spearman r coefficient is: 0.97 (PCS) and 0.89 (MCS). For age groups, respectively: 66–70: 0.97 (PCS) and 0.89 (MCS); 71–75: 0.97 (PCS) and 0.92 (MCS); 76–80: 0.96 (PCS) and 0.86 (MCS); >80: 0.96 (PCS) and 0.90 (MCS). In the 66–70 and 71–75 age groups, a strong correlation was found between RF–ILQ and BP–ILQ. Spearman r coefficients were respectively: 0.86 and 0.81 in the age group of 66–70 and 0.82, and 0.84 in the age group of 71–75. In the group of respondents aged 76 to 80, the highest significant correlation was recorded between RF and ILQ (r = 0.85), BP and ILQ (r = 0.75) and MH and ILQ (r = 0.75). For respondents over 80 years of age, significant correlations were found between BP and ILQ (r = 0.90) and MH and ILQ (r = 0.72). In the PF sphere, a weak correlation with ILQ was noted in the youngest study group (66–70: r = 0.19). In the age group of 71–75, the correlation was higher and amounted to 0.49, while in the other two age groups, the correlation is weaker (76–80: r = 0.43; >80: r = 0.30). Detailed values of the Spearman r coefficient for the correlation are presented in Table A1, Table A2, Table A3, Table A4 and Table A5.

**Table A1:** Correlations of SF-36 spheres for all groups (Spearman’s r coefficient).

Spheres	Spheres											
	PF	RF	BP	GH	VT	SF	RE	MH	SCH	PCS	MCS	ILQ
PF		0.39	0.25	0.33	0.36	0.25	0.18	0.18	0.11	0.39	0.29	0.37
RF	0.39		0.56	0.58	0.33	0.49	0.42	0.49	0.32	0.92	0.62	0.86
BP	0.25	0.56		0.55	0.26	0.50	0.50	0.63	0.40	0.80	0.70	0.81
GH	0.33	0.58	0.55		0.44	0.59	0.53	0.39	0.40	0.67	0.69	0.71
VT	0.36	0.33	0.26	0.44		0.36	0.25	0.13	0.21	0.43	0.31	0.40
SF	0.25	0.49	0.50	0.59	0.36		0.57	0.45	0.61	0.62	0.74	0.69
RE	0.18	0.42	0.50	0.53	0.25	0.57		0.50	0.59	0.56	0.73	0.65
MH	0.18	0.49	0.63	0.39	0.13	0.45	0.50		0.48	0.62	0.88	0.75
SCH	0.11	0.32	0.40	0.40	0.21	0.61	0.59	0.48		0.50	0.63	0.57
PCS	0.39	0.92	0.80	0.67	0.43	0.62	0.56	0.62	0.50		0.77	0.97
MCS	0.29	0.62	0.70	0.69	0.31	0.74	0.73	0.88	0.63	0.77		0.89
ILQ	0.37	0.86	0.81	0.71	0.40	0.69	0.65	0.75	0.57	0.97	0.89	

PF—physical functioning; RF—role: physical; BP—bodily pain; GH—general health; VT—vitality; SF—social functioning; RE—role: emotional; MH—mental health; SCH—Subjective sense of change in health; PCS—physical component summary; MCS—mental component summary; ILQ SF-36—index of life quality.

**Table A2:** Correlations of SF-36 spheres for the age group of 66–70 years (Spearman’s r coefficient).

Spheres	Spheres											
	PF	RF	BP	GH	VT	SF	RE	MH	SCH	PCS	MCS	ILQ
PF		0.17	0.12	0.28	0.41	0.22	0.12		0.15	0.22	0.12	0.19
RF	0.17		0.52	0.48	0.16	0.43	0.33	0.45	0.34	0.88	0.54	0.82
BP	0.12	0.52		0.53	0.14	0.46	0.52	0.67	0.45	0.83	0.75	0.84
GH	0.28	0.48	0.53		0.39	0.58	0.44	0.31	0.44	0.62	0.61	0.64
VT	0.41	0.16	0.14	0.39		0.29	0.22		0.26	0.29	0.18	0.25
SF	0.22	0.43	0.46	0.58	0.29		0.47	0.40	0.61	0.58	0.68	0.64
RE	0.12	0.33	0.52	0.44	0.22	0.47		0.50	0.59	0.53	0.70	0.62
MH		0.45	0.67	0.31		0.40	0.50		0.50	0.63	0.90	0.76
SCH	0.15	0.34	0.45	0.44	0.26	0.61	0.59	0.50		0.57	0.67	0.63
PCS	0.22	0.88	0.83	0.62	0.29	0.58	0.53	0.63	0.57		0.77	0.97
MCS	0.12	0.54	0.75	0.61	0.18	0.68	0.70	0.90	0.67	0.77		0.89
ILQ	0.19	0.82	0.84	0.64	0.25	0.64	0.62	0.76	0.63	0.97	0.89	

PF—physical functioning; RF—role: physical; BP—bodily pain; GH—general health; VT—vitality; SF—social functioning; RE—role: emotional; MH—mental health; SCH—subjective sense of change in health; PCS—physical component summary; MCS—mental component summary; ILQ SF-36—index of life quality.

**Table A3:** Correlations of SF-36 spheres for the age group of 71–75 years (Spearman’s r coefficient).

Spheres	Spheres											
	PF	RF	BP	GH	VT	SF	RE	MH	SCH	PCS	MCS	ILQ
PF		0.48	0.34	0.47	0.36	0.40	0.42	0.29	0.21	0.49	0.45	0.49
RF	0.48		0.50	0.61	0.31	0.61	0.56	0.42	0.43	0.90	0.62	0.83
BP	0.34	0.50		0.64	0.25	0.64	0.64	0.69	0.52	0.80	0.77	0.83
GH	0.47	0.61	0.64		0.43	0.72	0.64	0.46	0.48	0.74	0.76	0.78
VT	0.36	0.31	0.25	0.43		0.34	0.30		0.19	0.41	0.29	0.38
SF	0.40	0.61	0.64	0.72	0.34		0.66	0.60	0.67	0.77	0.85	0.83
RE	0.42	0.56	0.64	0.64	0.30	0.66		0.59	0.57	0.70	0.79	0.77
MH	0.29	0.42	0.69	0.46		0.60	0.59		0.54	0.61	0.88	0.75
SCH	0.21	0.43	0.52	0.48	0.19	0.67	0.57	0.54		0.62	0.67	0.66
PCS	0.49	0.90	0.80	0.74	0.41	0.77	0.70	0.61	0.62		0.81	0.97
MCS	0.45	0.62	0.77	0.76	0.29	0.85	0.79	0.88	0.67	0.81		0.92
ILQ	0.49	0.83	0.83	0.78	0.38	0.83	0.77	0.75	0.66	0.97	0.92	

PF—physical functioning; RF—role: physical; BP—bodily pain; GH—general health; VT—vitality; SF—social functioning; RE—role: emotional; MH—mental health; SCH—subjective sense of change in health; PCS—physical component summary; MCS—mental component summary; ILQ SF-36—index of life quality.

**Table A4:** Correlations of SF-36 spheres for the age group of 76–80 years (Spearman’s r coefficient).

Spheres	Spheres											
	PF	RF	BP	GH	VT	SF	RE	MH	SCH	PCS	MCS	ILQ
PF		0.56	0.28	0.23	0.29			0.29		0.48	0.24	0.43
RF	0.56		0.54	0.56	0.40	0.33	0.26	0.52	0.19	0.91	0.57	0.85
BP	0.28	0.54		0.53	0.33	0.35	0.31	0.47	0.23	0.78	0.53	0.75
GH	0.23	0.56	0.53		0.39	0.49	0.47	0.43	0.37	0.66	0.67	0.70
VT	0.29	0.40	0.33	0.39		0.33	0.18	0.30	0.18	0.52	0.38	0.49
SF		0.33	0.35	0.49	0.33		0.64	0.40	0.61	0.48	0.72	0.59
RE		0.26	0.31	0.47	0.18	0.64		0.41	0.69	0.41	0.69	0.54
MH	0.29	0.52	0.47	0.43	0.30	0.40	0.41		0.39	0.59	0.88	0.75
SCH		0.19	0.23	0.37	0.18	0.61	0.69	0.39		0.39	0.58	0.49
PCS	0.48	0.91	0.78	0.66	0.52	0.48	0.41	0.59	0.39		0.70	0.96
MCS	0.24	0.57	0.53	0.67	0.38	0.72	0.69	0.88	0.58	0.70		0.86
ILQ	0.43	0.85	0.75	0.70	0.49	0.59	0.54	0.75	0.49	0.96	0.86	

PF—physical functioning; RF—role: physical; BP—bodily pain; GH—general health; VT—vitality; SF—social functioning; RE—role: emotional; MH—mental health; SCH—subjective sense of change in health; PCS—physical component summary; MCS—mental component summary; ILQ SF-36—index of life quality.

**Table A5:** Correlations of SF-36 spheres for the age group of >80 years (Spearman’s r coefficient).

Spheres	Spheres											
	PF	RF	BP	GH	VT	SF	RE	MH	SCH	PCS	MCS	ILQ
PF		0.28	0.27		0.24			0.24		0.31	0.24	0.30
RF	0.28		0.59	0.52	0.44	0.49	0.51	0.53	0.32	0.95	0.69	0.90
BP	0.27	0.59		0.30	0.33	0.39	0.37	0.51	0.23	0.72	0.55	0.70
GH		0.52	0.30		0.48	0.38	0.48	0.22	0.16	0.52	0.57	0.57
VT	0.24	0.44	0.33	0.48		0.40	0.31	0.23		0.53	0.42	0.51
SF		0.49	0.39	0.38	0.40		0.49	0.30	0.51	0.59	0.65	0.64
RE		0.51	0.37	0.48	0.31	0.49		0.45	0.48	0.58	0.75	0.67
MH	0.24	0.53	0.51	0.22	0.23	0.30	0.45		0.46	0.59	0.83	0.72
SCH		0.32	0.23	0.16		0.51	0.48	0.46		0.46	0.56	0.51
PCS	0.31	0.95	0.72	0.52	0.53	0.59	0.58	0.59	0.46		0.77	0.96
MCS	0.24	0.69	0.55	0.57	0.42	0.65	0.75	0.83	0.56	0.77		0.90
ILQ	0.30	0.90	0.70	0.57	0.51	0.64	0.67	0.72	0.51	0.96	0.90	

PF—physical functioning; RF—role: physical; BP—bodily pain; GH—general health; VT—vitality; SF—social functioning; RE—role: emotional; MH—mental health; SCH—subjective sense of change in health; PCS—physical component summary; MCS—mental component summary; ILQ SF-36—index of life quality.
